# Modified Acrylate Pressure-Sensitive Adhesives for Low-Surface-Energy Substrate and Adhesion Mechanism Models

**DOI:** 10.3390/polym17091130

**Published:** 2025-04-22

**Authors:** Lucheng Shi, Haoran Shi, Jun Qian, Yifeng Shi

**Affiliations:** 1 Key Laboratory of Specially Functional Polymeric Materials and Related Technology (Ministry of Education), School of Materials Science and Engineering, East China University of Science and Technology, Shanghai 200237, China; lulushia@163.com (L.S.); 17664151850@163.com (H.S.); 2 Shanghai Hongdingfang Science Co., Ltd., Shanghai 200237, China; yifeng.shi@139.com

**Keywords:** pressure-sensitive adhesive, low-surface-energy substrate, hydroxylated polybutadiene, peel, adhesion mechanism

## Abstract

Most acrylate adhesives do not bond well to low-surface-energy substrates (e.g., polyethylene and polypropylene) due to the weak interaction force between the polar adhesive molecules and the substrate. To enhance the adhesion performance on low-surface-energy substrates and investigate the effects of substrate surface energy, roughness, pressure-sensitive adhesive (PSA) surface energy, viscosity, and modulus on adhesion performance, this study modifies the acrylate adhesive by incorporating a hydrogenated-terminated hydroxylated polybutadiene (HHTPB) structure with a double bond at one end. The results demonstrate an enhancement in the adhesion performance of the modified PSAs on High-Density Polyethylene (HDPE). The 24 h peel strength and loop tack increase to 4.88 N/25 mm and 8.14 N/25 mm at 20 °C, respectively, with the failure modes remaining adhesive failure. However, as the temperature increases, the peel strength decreases. The high-temperature resistance of the adhesive improves. Based on the experimental data, a mathematical model is proposed that incorporates both the wetting area and loss factor to predict peel strength. The influence of these two factors on the peel strength of the PSA is dependent on the application temperature of the adhesive.

## 1. Introduction

Adhesives are essential products in daily life and are widely used in various industries, such as medical, automotive, electronics, and packaging [1,2,3,4]. Acrylate pressure-sensitive adhesive (APSA) is a viscous polymer synthesized through the polymerization of acrylate monomers. It is one of the most widely used types of pressure-sensitive adhesives (PSA) due to its unique properties. APSA is a viscoelastic material that exhibits both liquid and solid properties. It combines the fluidity and wettability of a liquid with the cohesive strength of a solid. Particularly on polar and porous surfaces, APSA demonstrates excellent adhesion properties and remarkable aging resistance [5,6,7,8]. The factors affecting the adhesion properties of PSA are mainly viscosity, modulus, surface energy of PSA and the surface energy, roughness of substrate [9].

Low-surface-energy materials primarily include polyolefins, such as polyethylene (PE), polypropylene (PP), polybutadiene (PB), and fluorine-containing polymers like polytetrafluoroethylene. These materials are low-surface-energy substrates, and their surfaces can only form weak dispersion forces, lacking orientation and induced forces [10,11]. Common acrylate adhesives have high polarity and weak interaction forces with non-polar surfaces. Additionally, most common acrylate adhesives exhibit poor wetting performance on low-surface-energy surfaces, making it difficult for them to spread evenly on non-polar surfaces, which results in reduced adhesive strength [12].

At present, improving the adhesion performance of non-polar surfaces can be divided into the following two aspects: one is to carry out surface treatment of low-surface-energy substrates, and the second is to improve the adhesive bonding capacity of the adhesives [13,14]. There are many non-polar surface treatment methods, mainly chemical reagent treatment [15], flame treatment [16], corona treatment [17], and plasma treatment [18]. These surface treatment methods can enhance surface polarity, surface energy, and roughness while also eliminating the weak interfacial layer on the material’s surface, thereby improving bonding performance. However, on the other hand, they can also damage the properties of the substrate itself, increase the process and production costs, and have less application in industrial production [19].

Therefore, considerable attention has been devoted to the research and development of ideal adhesives through direct modification of pressure-sensitive adhesives (PSAs). In the past decade, PSAs designed for non-polar surfaces have primarily been modified by incorporating long-chain structures [20,21], fluorine-containing [22,23], or silicone-containing macromonomers [24], among others. These approaches enhance the adhesive performance on low-surface-energy substrates without altering the substrate. Such modifications improve the compatibility between the adhesive and low-surface-energy materials, resulting in excellent bonding performance. However, much of the research on PSA modification for low-surface-energy substrate applications has focused primarily on the change in adhesion performance without delving into the underlying factors that influence adhesion, such as the surface energy and rheological properties of PSAs. Furthermore, there is limited research on the relative contributions of these factors to the bond performance. For instance, according to the derivation of the subsequent formula, the wetting area, which plays a crucial role in bonding to non-polar surfaces, is not solely determined by the introduction of low-surface-energy monomers or groups but is also influenced by viscosity. The respective contributions of these factors to the bonding of non-polar surfaces have yet to be fully explored.

In order to improve the adhesion performance of APSA on low-surface-energy substrates and to investigate the role of surface energy and rheological properties on the bonding performance, this paper selects hydrogenated hydroxyl-terminated polybutadiene (HHTPB), which is readily available, inexpensive, and non-toxic, as a long-chain polymer to modify acrylate pressure-sensitive adhesives. HHTPB by itself does not have the ability to polymerize with acrylate monomers; therefore, it needs to be esterified with phthalic anhydride first and then polymerized with glycidyl methacrylate through a ring-opening reaction to obtain HHTPB macromonomers containing double bond structures. The physical properties of the modified acrylic adhesives were analyzed using gel permeation chromatography (GPC) and dynamic mechanical analysis (DMA). The effects of the introduction of the long-chain structure of HHTPB on the mechanical properties of the pressure-sensitive adhesive were analyzed by testing its adhesion performance. Regression analysis of the data obtained from the experiments led to a mathematical model of the peel strength and the surface energy and roughness of the substrate, the surface energy of the PSA, and viscosity and modulus of several influencing factors, which can be derived from the specific role of each factor in the amount of adhesive strength as well as the main factors that affect the adhesion properties in different temperature ranges.

## 2. Materials and Methods

### 2.1. Materials

Phthalic anhydride (PA), p-toluenesulfonic acid (TsOH), and methylbenzoquinone (MBQ) were purchased from Shanghai Maikelin Biochemical Co., Ltd. (Shanghai, China). Glycidyl methacrylate (GMA), 2-ethylhexyl acrylate (2-EHA), ethyl acetate (EAc), vinyl acetate (VAc), acrylic acid (AA), 2,2′-azobis (2-methylpropionitrile) (AIBN), aluminum acetylacetonate (ALAA), and heptane were purchased from Sinopharm Chemical Reagent Co., Ltd. (Shanghai, China). Hydrogenated hydroxyl-terminated polybutadiene (HHTPB P2000 and HHTPB P3000) was supplied by Shanghai Hongdingfang Technology Co., Ltd. (Shanghai, China).

### 2.2. Synthesis of HHTPB Macromonomers

**Figure 1:**
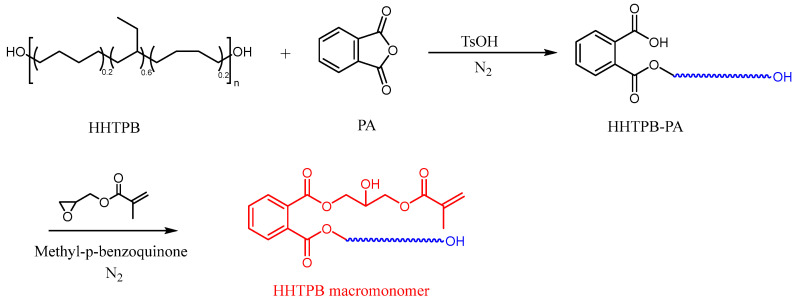
Synthesis of HHTPB macromonomers.

The reaction was carried out to synthesize HHTPB containing polymerizable double bonds through a two-step reaction. HHTPB was weighed and placed in a 500 mL four-necked flask. PA corresponding to the half molar mass and 0.12 wt% of the catalyst TsOH were added, and the mixture was stirred under nitrogen at a constant temperature of 120 °C for 9 h, resulting in a colorless transparent viscous liquid. A polymerization inhibitor, methyl benzoquinone (1 wt%), was weighed and dissolved in GMA. The mixed solution was then pumped into the flask, and the reaction was continued for 9 h under constant temperature nitrogen stirring at 115 °C, yielding a yellowish transparent viscous liquid. The mixture was poured out while hot, and an appropriate amount of heptane was added to dissolve the impurities. After filtration, the solution was purified to obtain a modified HHTPB-heptane solution. Heptane was removed under vacuum at 90 °C and −90 kPa, resulting in the HHTPB monomer, which was then sealed and stored. The preparation process of HHTPB macromonomers is shown in Figure 1.

### 2.3. Synthesis of HHTPB Modified Acrylic Adhesives

**Figure 2:**
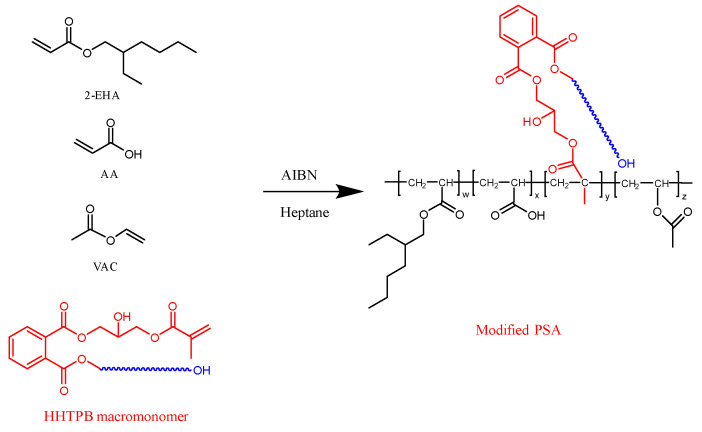
Synthesis of modified acrylate pressure-sensitive adhesives.

**Figure 3:**
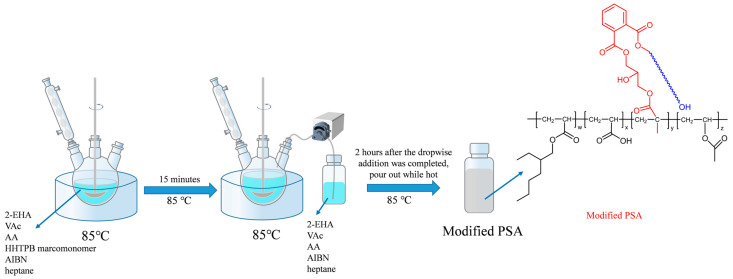
Schematic diagram of the preparation method of PSA.

The adhesives were prepared using 2-EHA as a soft monomer, VAc as a hard monomer, AA as a functional monomer, HHTPB macromonomers as modifiers, AIBN as an initiator, and heptane as the solvent. The molecular weights and contents of the HHTPB macromonomers in the different samples are listed in Table 1. The procedure was as follows: a portion of the reactive monomer, solvent, HHTPB macromonomers, and initiator were added to a four-necked flask with a mechanical stirring paddle and a condenser tube in a water bath at 85 °C. After stirring for 15 min, the remaining monomer, initiator, and solvent were added dropwise using a peristaltic pump. The modified acrylate pressure-sensitive adhesive was produced by holding the mixture for 2 h after the completion of dropwise addition. The polymerization equation for PSA is shown in Figure 2, and a schematic of PSA preparation is shown in Figure 3. The crosslinking agent ALAA and the solvent EAc were prepared into a crosslinking agent solution according to a mass ratio of 1:50, and the amount of crosslinking agent was adjusted to control the crosslinking degree of the PSAs. The configured crosslinking agent solution was added to each of the PSAs according to different qualities and stirred thoroughly with an electric stirrer to ensure complete homogeneous mixing. Then, PSA samples with different crosslinking degrees can be prepared [25,26].

### 2.4. Preparation of Pressure-Sensitive Adhesive Tape Samples

The crosslinked pressure-sensitive adhesive samples were formulated into a homogeneous adhesive solution with a solid content of 33%. Coating rods with a thickness of 100 μm were selected, and the adhesive was uniformly coated onto release paper. The coated samples were then dried in an oven at 130 °C for 5 min to remove any residual solvent. After cooling to room temperature, a PET film was applied, resulting in an adhesive film with a gram weight of approximately 25 g/m^2^. Finally, the adhesive film was cut into standard sample strips measuring 150 mm × 25 mm and 125 mm × 25 mm for subsequent testing.

### 2.5. Characterization

FT-IR spectra were obtained using a Magna-IR 550 infrared spectrometer (Thermo Nicolet Corporation, Waltham, MA, USA) to confirm the chemical structure of the HHTPB macromonomers. A small amount of the sample was thinly coated on a release paper, pressed uniformly, and used for testing. The spectral scanning range was set from 4000 to 400 cm^−1^.

The weight average molecular weight (Mw), number average molecular weight (Mn), and polydispersity index (PDI) of PSA were determined using a Waters e2695 gel permeation chromatograph (Waters Technology Co., Ltd., Milford, MA, USA). An appropriate amount of sample was completely dissolved in tetrahydrofuran(chromatography-grade) at a concentration of 1.5 mg/mL. The mobile phase was HPLC-grade tetrahydrofuran, and the stationary phase was polystyrene gel.

The storage modulus (G’), loss modulus (G”), complex viscosity (η) and loss factor (tanδ) were determined by dynamic thermomechanical analysis (DMA) using a HAAKE MARs 60 rheometer (Thermo Fisher Scientific, Waltham, MA, USA). During the test, the dried samples were heated under constant strain from −60 to 160 °C at a temperature increase rate of 6 °C/min.

The contact angle and surface energy at different temperatures were tested using a KJ-625 Water Drop Angle Tester (Guangdong Kejian Instrument Co., Ltd., Dongguan, China). The temperature of the hot bench was adjusted to the desired experimental temperature. The coated side of the adhesive film was then placed facing upward on the hot bench. A drop of liquid was slowly deposited onto the adhesive film, and the contact angle was measured. The liquid size was set to 2 μL. The contact angle of the film was measured by placing drops of liquid at different locations on the same film three times, and the average value was calculated. The surface energy test liquids were deionized water and ethylene glycol, and the surface energy was calculated using the OWRK method [27].

Peel strength was tested under the corresponding temperature conditions using a KJ-1067 testing machine (Guangdong Kejian Instrument Co., Ltd., Dongguan, China) with reference to the GB/T 2792-2014 standard [28], and the testing speed was 300 mm/min. Adhesive sample strips with dimensions of 150 mm × 25 mm were pasted on the HDPE and rolled five times with a 2 kg pressure roller. The tests were conducted after 20 min and 24 h. Each sample was tested three times, and the results were averaged.

The loop tack of the adhesives was tested using a KJ-6031 annular tack test machine (Guangdong Kejian Instrument Co., Ltd., Dongguan, China) with reference to the GB/T 31125-2014 standard [29]. An adhesive sample strip with a size of 125 mm × 25 mm was pasted on the HDPE and rolled five times with a 2 kg pressure roller to start the test. The instrument was used to raise the test at a speed of 300 mm/min. Each sample was tested three times, and the results were averaged.

## 3. Results and Discussion

### 3.1. Structural Characterization of Modified HHTPB

**Figure 4:**
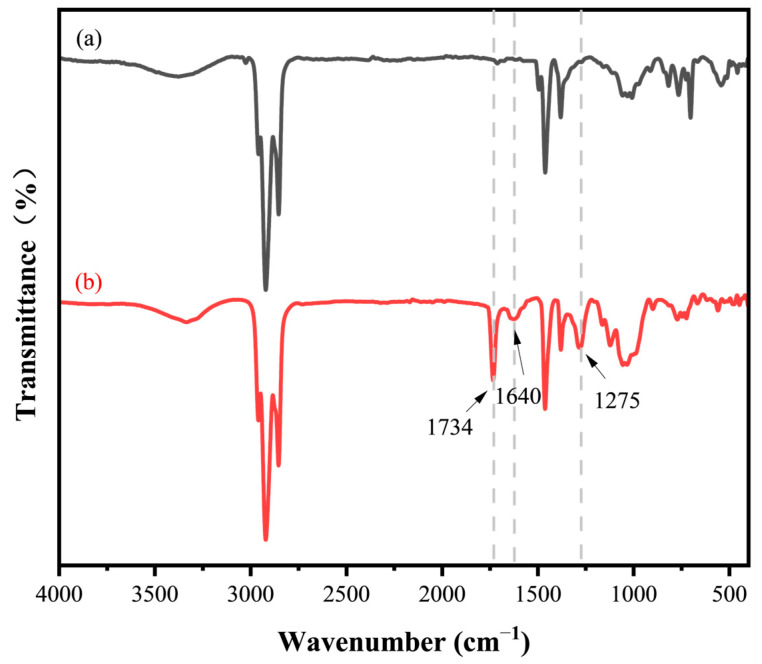
FT-IR spectra of HHTPB and synthesized HHTPB macromonomers: (**a**) HHTPB and (**b**) HHTPB macromonomers.

The synthesis of the HHTPB macromonomers was monitored using ATR-FTIR. As illustrated in Figure 4, the absorption peak at 1734 cm^−1^ for HHTPB macromonomers is the C=O bond, which proves the esterification reaction; the absorption peak at 1640 cm^−1^ is the C=C bond stretching vibration absorption peak, which proves the introduction of the polymerizable double bond; and 1275 cm^−1^ is the C-O bond stretching vibration absorption peak [28,29].

### 3.2. Molecular Weight Characterization

To study the effect of HHTPB macromonomers on PSA and to avoid the effect of a large difference in molecular weight, the number average molecular weight (Mn) of PSA was controlled to be similar in this work, and the molecular weights of PSA before and after modification were tested. All molecular weights are presented as average relative molecular weights. The results are presented in Table 2. The weight average molecular weight (Mw) and polydispersity index (PDI) of P_Null_ were significantly smaller than those of the modified PSAs. The introduction of a long-chain structure affects the rate of chain transfer and termination reactions, which makes the length distribution of the polymer chain more in homogeneous, thus increasing the PDI. Mw is more sensitive to high molecular weight portions, and the increase in the Mw of modified PSA reflects the contribution of long-chain macromonomers to the polymer and the addition of long-chain macromonomers.

### 3.3. Dynamic Mechanical Analysis

**Figure 5:**
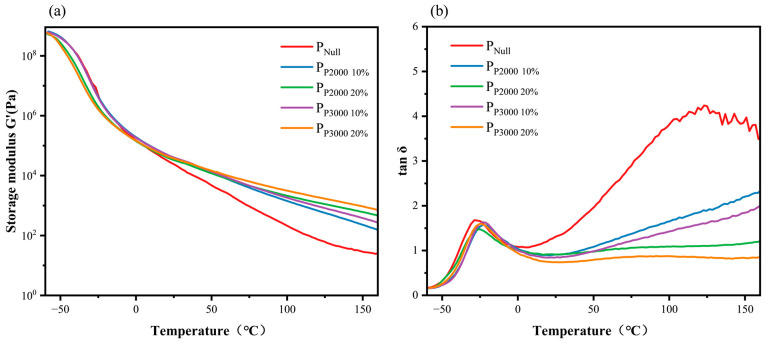
(**a**) Storage modulus (**b**) tanδ curves of PSAs.

PSA is a viscoelastic material. To further characterize the viscoelasticity properties of the modified PSAs under dynamic external stress, DMA tests were conducted on the PSAs before and after modification in the temperature range of −60–160 °C. Plots of the storage modulus (G’) and loss factor (tanδ) with respect to temperature were obtained, as well as their glass transition temperatures (Tg), as shown in Table 3 and Figure 5.

The main component of all five groups of PSAs is the soft monomer 2-EHA, which has a low Tg at about −70 °C. Therefore, the overall Tg of PSA is low, below −20 °C, as shown in Table 3. The long chain has a tangling effect, and its addition restricts the free movement of the acrylate molecular chain. The movement of the molecular chain segments requires higher energy and temperature, leading to an increase in Tg [30]. Due to the same formulation, only changing the molecular weight of the modified HHTPB macromonomer and the amount of addition, there is little difference between the Tg of the four modified PSAs.

As shown in Figure 5a, the storage modulus (G’) of the different PSAs all decrease sharply in the transition state region near Tg, and the decreasing trend slows when the temperature exceeds the transition state region. Compared with the low-temperature section, the storage modulus in the high-temperature section is very low, and in combination with the tanδ curve in Figure 5b, it can be assumed that this phenomenon occurs because of the viscous flow of the adhesive sample in the high-temperature section. The storage modulus of the modified PSA is significantly larger than that of the unmodified PSA above 0 °C, and the higher the content of the HHTPB macromonomer, the relatively higher the storage modulus. Above 100 °C, the tanδ curves of the unmodified PSA are obviously jittery compared with those of the modified four groups of PSA curves, and it is no longer possible to maintain the conventional morphology of the PSA, which suggests that the modified PSA has a better temperature resistance performance. The modified PSA exhibits a lower tanδ above 0 °C due to the restricted freedom of movement of the molecular chains, indicating that its energy dissipation is reduced and it is more inclined to the elastomer form [31].

**Figure 6:**
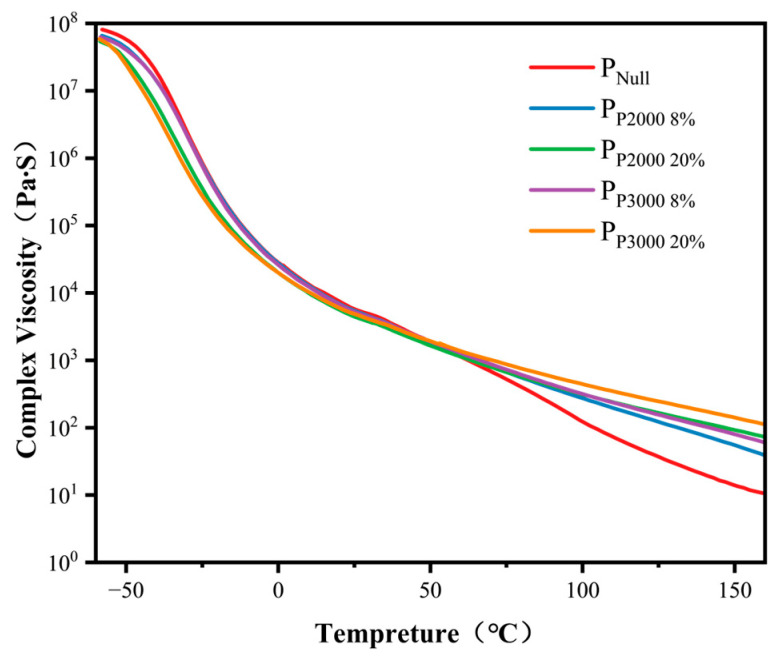
The complex viscosity (η) of the PSAs.

Figure 6 shows the curves of the complex viscosity of the five groups of PSA with temperature, and Table 4 lists the complex viscosity of PSA at different temperatures. As shown in Figure 6, the overall trend of the complex viscosity of the five groups of PSA is approximately the same, which is due to the fact that the main body formulation is the same, and only changing the content of modified macromonomers has little effect on the viscosity. At temperatures below 60 °C, the viscosities of the five sample groups are similar. The two groups of PSAs with the highest content of HHTPB long chains exhibit relatively low viscosity, while P_Null_ exhibits the highest viscosity. This can be attributed to the fact that long-chain molecules are able to slide more easily, and, compared to shorter molecules, they are less likely to form strong network structures or intermolecular cross-links, which results in a reduction in the overall viscosity of the fluid. The decreasing trend of the P_Null_ viscosity is more obvious than that of the remaining four groups of PSA above 60 °C. At high temperatures, the polymer movement becomes more active; however, the long-chain molecules, due to their larger spatial structure, tend to be more difficult to completely unravel or “curl”, thus maintaining a certain degree of intermolecular forces and structural stability, which results in a slower decrease in viscosity.

### 3.4. Surface Energy Analysis

The adhesion properties of PSA are related to its own surface energy and that of the substrate. The surface energy of the substrate at different temperatures was tested, and the results are listed in Table 5. The surface energy of HDPE (γ_S_) decreases with increasing temperature in this interval, but this change is not significant. As the temperature increases, the flexibility of the HDPE molecular chain increases and the thermal motion is intensified, facilitating the rearrangement of the surface molecules. At low temperatures, the molecular arrangement on the HDPE surface is relatively dense, while at higher temperatures, the molecular spacing increases, leading to a decrease in surface energy.

**Figure 7:**
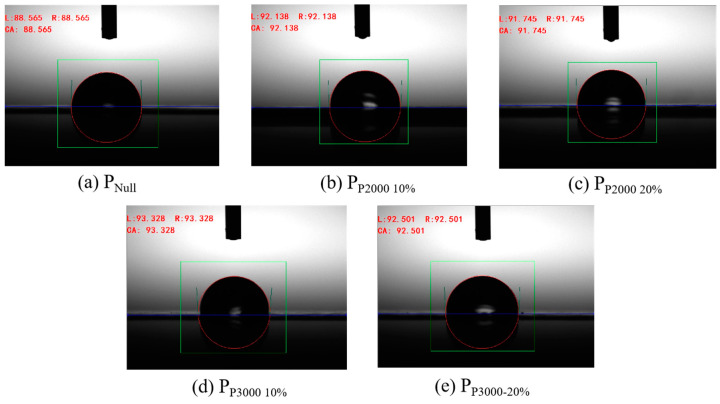
Contact Angle of PSAs at 20 °C, (**a**) P_Null_, (**b**) P_P2000 10%_, (**c**) P_P2000 20%_, (**d**) P_P3000 10%_, (**e**) P_P3000-20%_.

The water contact angle of PSA at different temperatures and its surface energy (γ_L_) were tested, as shown in Figure 7 and Table 6 and Table 7, which show that the contact angle between all PSAs and water became significantly smaller with increasing temperature. The increase in temperature led to the enhancement of molecular thermal movement and the weakening of the intermolecular interaction force, which made it easy for the water molecules to wet the surface of the adhesive film [32]. Correspondingly, the surface energy of each PSA sample also decreased with increasing temperature.

### 3.5. Adhesion Performance

**Figure 8:**
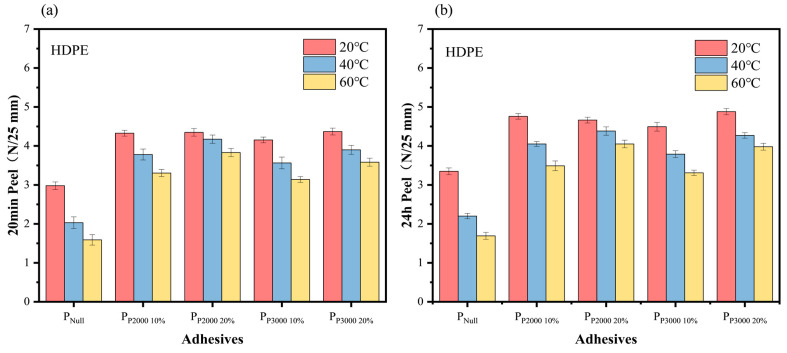
The 20 min (**a**) and 24 h (**b**) 180° peel strengths of PSAs on HDPE at different temperatures, all the destruction modes are adhesive failure.

Peel. In order to compare the effect of the introduction of HHTPB macromonomers on the adhesion properties of PSA on low-surface-energy surfaces, HDPE was selected as the low-surface-energy substrate, and the 20 min and 24 h peel strengths of PSA corresponding to different modified HHTPB monomer additions on HDPE sheets were determined. The test results are presented in Figure 8.

The introduction of HHTPB macromonomers did not change the destruction mode of the PSAs, which could be cleanly peeled off from the surface of the substrate. The introduction of the long-chain structure of HHTPB resulted in a higher peel strength at all three temperatures compared to the unmodified PSA. The high storage modulus of the modified PSA renders it less prone to deformation during peeling, leading to higher stress concentrations and, thus, higher peel strength. The non-polar side groups on the polymer chains weakened the intermolecular chain forces, which reduced the cohesion of the polymer solution and improved its adhesion to the low-surface-energy substrates. With an increase in bonding time, the wetting area increases, and the polymer molecular chains have sufficient time to move slowly to the surface of the substrate to form a strong adhesion; thus, the 24 h peel strengths are all higher than the 20 min peel strengths. As shown in the DMA results (Figure 5a), the increase in temperature significantly decreases the PSA storage modulus, weakens the interaction force between its molecular chains, decreases the cohesion strength and the viscosity, and the strength of resistance to external forces. Therefore, the peel strength of the five groups of PSAs decreased with increasing temperature [33,34].

**Figure 9:**
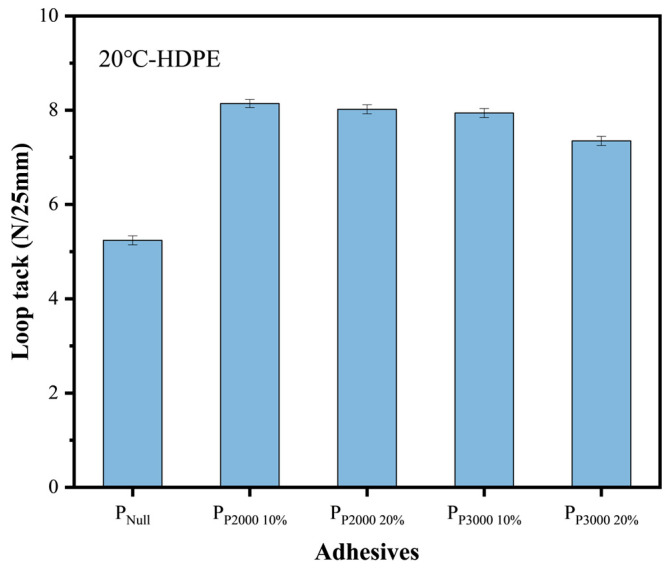
The loop tack of PSAs on HDPE, all the destruction modes are adhesive failure.

Loop tack. The loop tack of the PSAs was tested to further evaluate the adhesion performance of the modified PSAs. The results are shown in Figure 9. The incorporation of HHTPB macromonomers improves the loop tack of PSAs on HDPE. As demonstrated by the DMA test (Figure 5), the modified PSA exhibits more elastomeric behavior and maintains a certain degree of deformability under pressure, reducing the likelihood of a delamination. Additionally, the increased viscosity results in a less fluid adhesive layer, which was better able to maintain contact with the substrate surface during the initial contact. This improved wetting behavior increases the contact area between the adhesive and substrate, thereby enhancing the loop tack.

### 3.6. Influencing Factors of Peel Strength

Peeling behavior is related to the modulus of the PSA and wetting area, while the wetting area is related to the surface energy of the adhesive, viscosity of the adhesive and surface energy and roughness of the substrate.

Wetting in adhesives refers to the phenomenon of a liquid adhering to a solid surface when the PSA comes into contact with the substrate. Since adhesion involves contact of the whole surface, the wetting at this time is different from the wetting phenomenon of an isolated droplet on the surface, which refers to the wetting within the pores of the rough surface. Different wetting abilities can change the wetting area, which will have an effect on the subsequent peeling behavior. The final degree of wetting is thermodynamically controlled, while the process of reaching the final degree of wetting is controlled by kinetic factors [35,36].

T. Young proposed Young’s equation [37] through the mechanical analysis method; based on Young’s equation, Neumann proposed the ES second state equation [38], which can be used for the explanation of bonding behavior; the equation is as follows:

(1) $$\gamma_{SL}=\frac{{\left(\sqrt{\gamma_{S}}-\sqrt{\gamma_{L}}\right)}^{2}}{1-0.015\sqrt{\gamma_{S}\gamma_{L}}}$$

In Equation (1), *γ_SL_*, *γ_S_* and *γ_L_* represent the interfacial tension, surface energy of the substrate, and surface energy of the PSA, respectively. Conversely, the basic prerequisite for the formation of PSA bonding is that the PSA can wet the substrate surface. Wetting is divided into two processes: (1) spreading on the surface of the substrate and (2) penetration into the substrate surface cavities and formation of a specific adhesive surface, i.e., the PSA must be able to form a bond on the surface of the fully unfolded adhesive substrate so that the molecules of the PSAsurface and substrate are in close contact to form an intermolecular adsorption force [39].

The actual substrate is seldom completely smooth. If the pores on the sticky substrate are regarded as capillary tubes, the velocity of a liquid with viscosity (*η*) at time (*t*) deep into a certain capillary tube with radius (*R*) and length (*L*) can be calculated according to the following equation:

(2) $$\frac{dL}{dt}=\frac{{(\gamma }_{S}-\gamma_{SL})\times R}{4\eta L}=\frac{R\gamma_{L}cos\theta }{4\eta L}$$

By integrating the above equation, we obtain:

(3) $$t=\frac{2\eta L^{2}}{R{(\gamma }_{S}-\gamma_{SL})}=\frac{2\eta L^{2}}{R\gamma_{L}cos\theta }$$

In Equation (3), *η* represents the viscosity of PSA, and *θ* represents the contact angle. Combining Young’s equation: $\gamma_{S}=\gamma_{SL}+\gamma_{L}cos\theta$ with Equations (1) and (3) can obtain Equation (4):

(4) $$t=\frac{2\eta L^{2}}{\left[\gamma_{L}-\frac{{\left(\sqrt{\gamma_{S}}-\sqrt{\gamma_{L}}\right)}^{2}}{1-0.015\sqrt{\gamma_{S}\gamma_{L}}}\right]R}$$

Within a single capillary pore, the wetting area of the PSA and capillary surfaces (*A*) is calculated using Equation (5):

(5) $$A=\sqrt{2\frac{\left[\gamma_{L}-\frac{{\left(\sqrt{\gamma_{S}}-\sqrt{\gamma_{L}}\right)}^{2}}{1-0.015\sqrt{\gamma_{S}\gamma_{L}}}\right]\pi^{2}R^{3}}{\eta }*t}$$

In Equation (5), the main factors affecting the variation in the wetting area (*A*) within a single capillary pore channel with time (*t*) are *γ_L_*, *γ_S_*, *η*, and *R*. The two surface energies of the PSAs have been measured in the calculation, as shown in Table 5 and Table 7. The viscosity of solid PSA is difficult to measure; therefore, complex viscosity is used instead of the viscosity to simulate the viscosity of different PSA at different temperatures, as shown in Table 4. R is determined to be 0.1 μm.

Combined with the PSA commonly used temperature range and test conditions, three testing temperatures: 20 °C, 40 °C, and 60 °C were selected. According to Equation (5) and the measured data mentioned above, the wetting areas of the PSAs on the capillary pore adhesive and capillary pore surface can be calculated. The time (*t*) is 24 h. The results are shown in Table 8. The calculated wetting area for the five groups of samples increases with temperature. This is attributed to the significant decrease in viscosity as the temperature increases, which enhances the fluidity of the PSA. As a result, the PSA spread more easily on the substrate surface, leading to a larger wetting area.

The wetting area (*A*), energy storage modulus (*G’*), loss modulus (*G”*), and loss factor (*tanδ*) are commonly used parameters to characterize the adhesion behavior of PSA. This work focuses on the results of the measured mechanical properties and explores the different mechanisms represented by the combinations of these four parameters. A data regression analysis was performed on the experimental data, and the corresponding peel strength (*P*) model and correlation coefficient (*R*) were obtained, as shown in Table 9.

Model (a) corresponds to a mechanism in which the peeling force is entirely controlled by the contact area (*A*) of the adhesive within the wetting aperture. However, the regression results of the measured data in this study show a correlation coefficient of only 0.147, indicating a weak correlation between the measured data and this mechanism. This suggests that the peeling force is not significantly correlated with the wetting area as a single factor, and other factors likely influence the peeling force in conjunction with the wetting area.

Model (b), which incorporates both the wetting area and the modulus as influencing factors, yields a higher correlation coefficient, indicating that the model effectively describes the peeling behavior. This suggests that the peeling force can be regulated by three factors: the storage modulus (*G’*), loss modulus (*G”*), and single capillary channel wetting area.

In Model (b), the storage modulus (*G’*) and loss modulus (*G”*) are closely related to each other. Therefore, by replacing the *G’* and *G”* terms with the loss factor (*tanδ*) in Model (c), a simplified model is obtained. The correlation coefficient of Model (c) remains greater than 0.9, demonstrating that it can effectively describe the peeling behavior.

Figure 5b shows that the loss factor (*tanδ*) in Model (c) is close to 1. This parameter represents a strength property that remains unaffected by changes in the wetting area. In both Models (b) and (c), item A refers to the wetting area within a single capillary channel. While it does not significantly contribute to the peeling force by itself, it is a breadth property that influences the peeling force through the additive and cumulative effects of numerous capillary channels. Since the peel strength is measured linearly, and assuming that the plate cavities are uniformly distributed and closely spaced, with a cavity radius of 1 × 10^−7^ m and a diameter of 2 × 10^−7^ m, the number of cavities along a line is calculated as 25 mm/(2 × 10^−7^ m), or 1.25 × 10^5^ cavities. These capillaries collectively contribute to the total wetting area. If A in Model (c) is replaced by the total wetting area, Model (c) can be rewritten as Equation (6), as follows:

(6) $$P=7.51/({1.25\times 10^{5})}^{0.03}\times ({1.25*10^{5}*A)}^{0.03}\times {tan\delta }^{-0.94}$$

Then:

(7) $$P=5.29\times (1.42*A^{0.03}){\times tan\delta }^{-0.94}$$

In Equation (7), $1.42\times A^{0.03}$ represents the total contribution wetting area.

By analyzing Equation (7), below 0 °C, the *tanδ* values of the five adhesives were approximately the same; therefore, the contribution of this parameter to the peel strength was also similar. However, the viscosity and surface energy of the unmodified PSA are higher, while the modified PSA exhibits a larger wetting area, which makes a more significant contribution to the peel strength of the PSA on HDPE. Between 0 °C and 60 °C, the difference in viscosity is relatively small, but the difference in surface energy is more pronounced. The loss factor of unmodified PSA ranges from 1 to 2, while that of modified PSAs fluctuates around 1 and is lower than that of unmodified PSA. In this temperature range, according to Model (c), both the loss factor and wetting area terms influence the peel strength, with the contribution of the loss factor term to the peel strength of the modified PSA still being greater than that of the unmodified PSA. Above 60 °C, the unmodified PSA exhibits higher viscosity and surface energy, contributing more to the peel strength of PSAs on HDPE. In contrast, the modified PSAs have a greater wetting area. At temperatures above 60 °C, the loss factor of the unmodified PSA is much higher than that of the modified PSAs, at which point the effect of the wetted area on the peel strength is significantly reduced and is primarily determined by the loss factor. Therefore, the adhesive strength of PSAs is jointly influenced by the wetting area and loss factor, with the wetting area being more affected by viscosity than the loss factor. The factors that primarily govern the adhesive strength of PSAs vary across different temperature ranges.

## 4. Conclusions

The adhesion performance of pressure-sensitive adhesives on low-surface-energy substrates was investigated, with a focus on several factors influencing peel strength, including substrate surface energy and roughness, as well as the surface energy, viscosity, and modulus of the PSAs. These factors were experimentally examined to determine their contributions to peel strength. The polymerizable double bond functionality of HHTPB macromonomers was achieved and used to modify the acrylate pressure-sensitive adhesive. The specific conclusions are as follows:

(1) The molecular weight and polydispersity index of PSA increase with increasing content of HHTPB. The glass transition temperature and storage modulus of the modified PSA improve, indicating enhanced high-temperature resistance and more elastomeric properties.
(2) The surface energy of the PSAs decreases upon the incorporation of the long-chain structure and further decreases with an increase in temperature. However, the effects of HHTPB macromonomers content and molecular weight on the surface energy of the PSAs are minimal.
(3) The incorporation of HHTPB macromonomers into the PSA enhances its adhesion properties on low-surface-energy substrates. The 24 h peel strength and loop tack increase to 4.88 N/25 mm and 8.14 N/25 mm at 20 °C, respectively, with the failure modes remaining adhesive failure.
(4) A regression analysis was conducted to examine the factors affecting the peel strength, and a model was proposed that links the peel strength to the combined effects of the wetted area and loss factor. The dominant factors influencing peel strength vary across different temperature ranges.

## Figures and Tables

**Table 1:** Specific formula for PSA samples.

Sample	HHTPB	Modified HHTPB (wt%)
P_Null_	/	0
P_P2000 10%_	P2000	10
P_P2000 20%_	P2000	20
P_P3000 10%_	P3000	10
P_P3000 20%_	P3000	20

**Table 2:** Molecular weight and polydispersity of the PSAs.

Sample	P_Null_	P_P2000 10%_	P_P2000 20%_	P_P3000 10%_	P_P3000 20%_
Mn of PSAs (×10^4^)	12.2	13	11.1	12.6	10.7
Mw of PSAs (×10^4^)	21.9	32.5	35.2	29.7	30.9
polydispersity	1.8	2.5	3.2	2.3	2.9

**Table 3:** Tg Values of PSAs.

Sample	P_Null_	P_P2000 10%_	P_P2000 20%_	P_P3000 10%_	P_P3000 20%_
Tg (°C)	−28.6	−23.0	−25.0	−23.2	−24.6

**Table 4:** The complex viscosity (η) of PSA at different temperatures (Pa∙S).

Temperature (°C)	P_Null_	P_P2000-10%_	P_P2000-20%_	P_P3000-10%_	P_P3000-20%_
20	3658.9	6557.5	5405.4	6564.1	5550.3
40	1295.9	2981.4	2431.2	2944.8	2543.5
60	402.4	1177.4	1089.3	1267.8	1278.9

**Table 5:** The surface energy of HDPE (γ_S_) at different temperatures (mN/m).

Substrate	20 °C	40 °C	60 °C
HDPE	27.1	26.8	26.0

**Table 6:** Contact angles of PSAs at different temperatures (°).

PSA	20 °C	40 °C	60 °C
P_Null_	88.6	77.9	78.6
P_P2000 10%_	92.1	90.5	90.6
P_P2000 20%_	91.7	88.6	90.0
P_P3000 10%_	93.3	89.5	90.1
P_P3000-20%_	92.5	89.1	89.6

**Table 7:** The surface energy of PSAs(γ_L_) at different temperatures (mN/m).

PSA	20 °C	40 °C	60 °C
P_Null_	29.4	31.7	31.0
P_P2000-10%_	22.8	18.8	17.8
P_P2000-20%_	23.8	20.0	19.6
P_P3000-10%_	22.2	19.4	19.1
P_P3000-20%_	23.0	19.6	19.4

**Table 8:** Calculated wetting area of PSAs on the capillary pore adhesive and capillary pore surface at 24 h (1 × 10^−11^ m^2^).

PSA	20 °C	40 °C	60 °C
P_Null_	11.7	20.4	35.8
P_P2000-10%_	7.67	10.2	16.0
P_P2000-20%_	8.66	11.7	17.5
P_P3000-10%_	7.55	10.4	16.0
P_P3000-20%_	8.38	11.3	16.1

**Table 9:** Models of peel strength and correlation coefficients.

Model	Factor	Expression	R
(a)	*A*	$P=1.82\times A^{-0.03}$	0.147
(b)	*A*, *G’*, *G’’*	$P=4.67\times A^{0.04}\times G^{\prime \prime -0.84}\times G^{\prime 0.91}$	0.948
(c)	*A*, *tanδ*	$P=7.51\times {A^{0.03}\times tan\delta }^{-0.94}$	0.935

## Data Availability

The original contributions presented in this study are included in this article. Further inquiries should be directed to the corresponding author.
